# Integrating body movement into attractiveness research

**DOI:** 10.3389/fpsyg.2015.00220

**Published:** 2015-03-03

**Authors:** Bernhard Fink, Bettina Weege, Nick Neave, Michael N. Pham, Todd K. Shackelford

**Affiliations:** ^1^Institute of Psychology, University of Göttingen, Göttingen, Germany; ^2^Courant Research Center Evolution of Social Behavior, University of Göttingen, Göttingen, Germany; ^3^Department of Psychology, Northumbria University, Newcastle upon Tyne, UK; ^4^Department of Psychology, Oakland University, Rochester, MI, USA

**Keywords:** attractiveness, body movement, dance, perception, personality, strength

## Abstract

People judge attractiveness and make trait inferences from the physical appearance of others, and research reveals high agreement among observers making such judgments. Evolutionary psychologists have argued that interest in physical appearance and beauty reflects adaptations that motivate the search for desirable qualities in a potential partner. Although men more than women value the physical appearance of a partner, appearance universally affects social perception in both sexes. Most studies of attractiveness perceptions have focused on third party assessments of static representations of the face and body. Corroborating evidence suggests that body movement, such as dance, also conveys information about mate quality. Here we review evidence that dynamic cues (e.g., gait, dance) also influence perceptions of mate quality, including personality traits, strength, and overall attractiveness. We recommend that attractiveness research considers the informational value of body movement in addition to static cues, to present an integrated perspective on human social perception.

## PERCEPTION OF PHYSICAL APPEARANCE

The morphology of the face and body affects people’s perceptions of others, and this has consequences for human social interaction (Fink and Penton-Voak, 2002; Grammer et al., 2003a; Gangestad and Scheyd, 2005; Rhodes, 2006). Physical appearance influences many social decisions, including consumer behavior, economic and recruitment decisions, and even legislation (Rhode, 2010; Hammermesh, 2011; Saad, 2011). Dion et al. (1972) first demonstrated that the assignation of desirable traits to attractive people (“what is beautiful is good”) serves as a cognitive heuristic deployed in social perception. Since this seminal study, researchers from several disciplines have investigated the causes of this assessment heuristic (for review, see Zebrowitz and Montepare, 2008). Evolutionary scientists argue that the human interest in facial and body morphology—and the social perceptions evoked by physical features—are neither arbitrary nor culture-bound, but instead reflect the operation of adaptations shaped by sexual selection (Grammer et al., 2003a,b; Little et al., 2011). These adaptations motivate the search for desirable qualities in a potential romantic partner (Buss, 1989, 1994; Etcoff, 1999). Although the evidence obtained from studies of faces and bodies supports this evolutionary perspective on attractiveness perceptions, studies of static cues capture a limited range of those present in social interaction.

Attractiveness research has relied heavily on the use of static representations (i.e., images) of the human face and body, thus neglecting the fact that in social situations we rarely observe others in this condition. In a meta-analysis, Langlois et al. (2000) concluded that there is variability in the results of attractiveness research with regard to the use of stimuli types (i.e., photographs, videos, and *in situ* encounters) and that this variability could produce different attractiveness judgments. With regard to face perception however, recent research does not confirm this assertion. For example, Koscinski (2013) reported a large positive correlation between attractiveness perceptions of static images and videos of the same faces, indicating that results of studies relying on attractiveness assessments of static images are ecologically valid. Rhodes et al. (2011) arrived at a similar conclusion, suggesting that the validity of static and dynamic images of faces in attractiveness research might result from the tendency to make rapid and robust attributions. There is evidence that people make trait inferences from physical appearance in milliseconds. In five experiments on assessments of unfamiliar faces, varying only in exposure time, Willis and Todorov (2006) demonstrated that judgments made after a 100 ms exposure correlated highly with judgments made in the absence of time constraints. However, it was also found that increased exposure time led to more differentiated person impressions. With increased exposure time, observers made a more fine-grained discrimination of trait attributes such that the effect of attractiveness on trait judgment was smaller.

## PERCEPTION OF BODY MOVEMENT

Rapid trait attribution has also been documented for perceptions of others’ body movements. Although the time required to accurately assess another person based on movement cues is not yet known, one can speculate that it is longer than that reported for face perception. Thoresen et al. (2012) presented point-light walkers to participants and identified the movement components that accounted for personality judgments. Observers made reliable trait judgments using a small number of movement components. Moreover, systematic manipulations of these components affected people’s judgments, indicating that certain movement cues (or combinations of them) influence personality perception. Observers were not necessarily accurate in their assessments however, as movement components were associated with perceived, but not with self-reported personality. Further analyses indicated that accuracy of trait ratings was accounted for by observers’ perceptions of emotion, attractiveness, and masculinity of the walkers. These results corroborate that of previous studies, and provide evidence that observers can identify basic emotions from body movement even when information about body morphology is impoverished, through—for example—the use of point-light displays (e.g., Atkinson et al., 2004; Clarke et al., 2005).

Most studies on the perception of human body movement have used visual representations of human walk (predominantly point-light displays) in their experimental paradigm. A rare exception is Dittrich et al. (1996), which showed that the conclusions about social perception derived from studies of human walk also apply to dance. These researchers instructed professional dancers to convey specific emotions (i.e., anger, disgust, fear, grief, joy, surprise) via body movements, which were captured and transferred into point-light displays. Participants’ recognition rate of the displayed emotions from point-lights was high (63% correct) and greater when fully lit videos of the dancers were used as stimuli (88% correct). Similar recognition rates have been found for human gait. Troje et al. (2005) presented point-light stimuli of human walkers from different viewpoint angles to male and female observers. The stimuli were altered such that observers viewed the normal (unaltered) walk, walks in which all stimuli had been normalized with respect to body size or body shape, and two conditions in which walking frequency was altered. After an initial presentation in which observers viewed the stimuli and were told the names of the walkers, they then received a series of training sessions. Recognition performance reached 90% after five training sessions. Observers accurately identified an individual when the stimuli had been normalized for body shape and walking frequency, leading the researchers to conclude that structural information (i.e., body morphology) plays a secondary role to gait kinematics in the identification of a person. This finding provides insight into the relative significance of certain cues (static and/or dynamic cues) in social perception by emphasizing the role of dynamic cues.

The ability to recognize emotions of conspecifics from their body movements, however, does not necessarily inform us about people’s preferences for certain types of body movement, and how such preferences may affect the way individuals form impression about others, including decisions about potential romantic partners. Mate selection forms an integral part of the sociocultural world, and as such we might expect that the sensitivity to movement cues extends to other features derived from movement, such as gender identity, sexual orientation, health, and personality. With regard to sexual orientation, for example, Johnson et al. (2007) presented computer-generated animations of humanoid characters (avatars) that varied morphologically (five levels of waist-to-hip ratio, WHR; Singh, 2002, 2006) and dynamically (five levels of motion ranging from extreme shoulder swagger to extreme hip sway). Judgments of sexual orientation of walkers perceived to be male were strongly affected by motion, but not morphology, whereas judgments of sexual orientation of walkers perceived to be female were affected by motion and morphology.

## DANCE AND INTERPERSONAL ATTRACTION

In the context of interpersonal attraction, studies investigating the role of body movement have focused on dance movements, perhaps because dance shows greater inter-individual variation than gait or running, and because dance is typically observed in courtship situations. Dance is a series of body movements composed of purposeful, intentional, rhythmical, and culturally patterned sequences that are part of the courtship practices of many cultures (Kurath, 1960; Kaeppler, 1978; Hanna, 1987). Hanna (1987, 2010) argued that dance is produced by sexually selected mechanisms designed to display beauty, health, strength, and sexual attractiveness. Recent research supports this hypothesis, as there is evidence that dance movements, particularly those of men, correlate with characteristics relevant to mate selection.

For example, Hugill et al. (2009) and McCarty et al. (2013) reported a positive relationship between women’s perceptions of the attractiveness and dance quality of men’s dances with the male’s physical strength (as measured via hand-grip strength). Recent research has confirmed the attractiveness-strength relationship in men, and reported its absence in women (Weege et al., in press b). Moreover, women’s assessments of men’s dance attractiveness were found to correlate with particular personality traits, including sensation-seeking (Hugill et al., 2011) and self-reported global personality descriptors (Fink et al., 2012b). Thus, there is evidence that, similar to the findings on associations of physical strength and male facial appearance (Fink et al., 2007), partner attractiveness and risk-taking behavior (Henderson et al., 2005), women make attributions about such qualities using cues found in men’s dance movements. Female sensitivity to these cues affects their mate preferences, as women judge dances of physically stronger men and those who score higher on sensation seeking to be more attractive. Hence men may convey information about their mate quality via dance movements^1^.

In the attempt to identify body movements that characterize a “good” male dancer, Neave et al. (2011) motion-captured dance movements of men dancing to a basic drumbeat. Women rated videos of shape-standardized humanoid avatars for perceived dance quality. “Good” dancers displayed larger and more variable bending and twisting movements of their head/neck and torso, and faster bending and twisting movements of their right knee. Given that comparative biological studies have suggested that females prefer vigorous and skilled males, Neave et al. (2011) concluded that women derive information about physical condition, including health, fitness, and genetic quality from male dancing ability. Weege et al. (2012) presented videos of pairs of “good” and “bad” dancers in an eye-tracking experiment and recorded dwell time and fixations of female participants, in addition to securing attractiveness and masculinity ratings for each dancer. “Good” male dancers received greater visual attention and were judged to be more attractive and masculine than “bad” male dancers. These results indicate a cognitive bias that facilitates mating-related motives [see Maner et al.’s (2003) “beauty captures the mind of the beholder” hypothesis].

Two studies investigated whether women’s body movements influence their attractiveness to men and, in particular, whether this relationship is mediated by female fertility. Using point-light displays, Provost et al. (2008) found that women at high-fertility (versus low-fertility) displayed gaits that were *less* attractive to men. In contrast, Fink et al. (2012a) used silhouette displays and found that women at high-fertility displayed gaits and dances that men rated as *more* attractive. Although it is unclear what might account for these conflicting results, there is independent evidence corroborating the findings from Fink et al. (2012a) For example, women at high-fertility (versus low-fertility) report increased sexual desire and wear more attractive clothing, and female lap dancers at high-fertility (versus low-fertility) report higher earnings (see Haselton and Gildersleeve, 2011). Thus, at high-fertility, women may alter their behavior—including the manner in which they walk and dance—to appear more attractive to men.

People derive information from body movements, particularly dance movements, and this affects attractiveness judgments and social perceptions. However, because most of this previous work included only European participants (with some bias toward the study of male dance), it is unknown whether the results generalize to other countries/societies. There is mixed evidence for claims regarding the universality of certain human features and displays. A recent example is facial expressions of emotions, which has been a matter of debate since Darwin’s (1872) seminal publication. For more than 100 years, it has been asserted that all humans communicate six basic emotional states using the same facial expressions/movements (Ekman and Friesen, 1971; Ekman et al., 1987). A recent study, however, refutes this hypothesis (Jack et al., 2012), showing that people from Western and Eastern cultures differ in their mental representations of the six emotions in terms of associated facial movements. There has been controversy about the Jack et al. (2012) study, with some scholars arguing that the findings do not refute the universality of facial expressions of emotions, but instead contribute to the investigation of perceptual consistencies and differences of specific emotional facial cues (Sauter and Eisner, 2013). A likely scenario is that evolved cognitive mechanisms and socio-cultural variables affect the perception of emotions from facial expressions, as has been reported for gestures and postures (Argyle, 1988; Moore et al., 2010). We suspect a similar interaction between evolved mechanisms and socio-cultural variables that also affects assessments of body movement.

## CROSS-CULTURAL PERCEPTION OF BODY MOVEMENT

Although cross-cultural similarities and differences in facial expressions of emotions have received attention from researchers in many disciplines (for review, see Russell et al., 2003), little research has focused on cross-cultural similarities and differences in perceptions of body movements. The fact that the ability to decode information from point-light animations of movement is done rapidly and accurately—and from an early age (Simion et al., 2008), and that this processing is served by dedicated neurological components (e.g., Grossman et al., 2000, 2005)—suggests that the detection and interpretation of biological motion may be produced by evolved cognitive mechanisms (Pica et al., 2011). There may be a “shared taste” in body movement perception across cultures, which is attributable to adaptations and which is independent of socio-cultural effects. If so, one might expect to find cross-cultural agreement in body movement perceptions, but empirical evidence on this hypothesis is scarce. Montepare and Zebrowitz (1993) investigated similarities and differences in American and Korean participants’ perceptions of age-related gait qualities in men and women. The researchers videotaped American men and women with reflective tape attached to major limb joints, highlighting movements against a black background. American and Korean observers judged the videos on several traits, including happiness, sexiness, and dominance. There was substantial cross-cultural agreement, even when controlling for perceived age and sex of the walkers. However, although American ratings of perceived dominance decreased with the walkers’ age, this relationship was not observed with Korean ratings. The researchers concluded that some judgments derived from gait might be universal, whereas others may be determined by culturally specific values that alter age-related gait cues.

More recently, Fink et al. (2014) recruited a sample of Brazilian and German women to view animated virtual characters of male dancers (for methodology, see Neave et al., 2011; Fink et al., 2012b). Women rated the attractiveness of 10 s videos of each dancer on a 5-point Likert-type scale. There was a significant positive correlation of Brazilian with German women’s assessments of men’s dance attractiveness, suggesting cross-cultural similarity in dance attractiveness perceptions. However, additional analyses considering the dancers’ personality (as assessed by the NEO-FFI; Costa and McCrae, 1992) as covariates revealed a significant difference between Brazilian and German women’s ratings of men’s dance movements, as well as significant interaction effects of country with two of the personality factors (neuroticism and conscientiousness).

These preliminary findings suggest that although there is some cross-cultural consensus in women’s perceptions of men’s dance movements, part of the variation in dance movement perception is attributable to culture-dependent personality cues (Schmitt et al., 2007) derived from dance movements. It is unclear, however, whether the attribution of certain personality characteristics to a dancer also corresponds with his/her “true” (i.e., self-reported) personality, or if personality attribution merely reflects a “halo” of attractiveness perception. The answer to this question is probably not solely in support of one of the two possibilities but rather a combination of the two. An interesting result in this context—though on face perception—is that of Little et al. (2006), who suggested that the attractiveness stereotype (*sensu* Dion et al., 1972) should be reconsidered in that attractiveness is not an unspecific attribution, but reflects the attribution of desired personality traits. In short, people provided higher attractiveness ratings when they thought that—based on facial appearance—the target has desired personality characteristics. We consider it likely that personality perception from dance movements may also be affected by personality characteristics desired in a romantic partner. Hence the inference to personality of a dancer via perceptions of dance attractiveness may be based on displays expressed in dance that do not match with (and are not caused by) self-reported personality.

Weege et al. (in press a) showed that men’s self-reported (NEO-FFI) personality scores did not correlate with women’s ratings of men’s personality standings, i.e., women were not able to accurately assess men’s personality from dance movements—a result that corroborates research investigating relationships of self-reported personality with observer-reports of personality based on gait (Thoresen et al., 2012). However, women’s ratings of a man’s dance attractiveness correlated negatively with their ratings of his neuroticism, and positively with their ratings of his conscientiousness. Weege et al. (in press a) suggested that there might be fundamental movement characteristics, which affect perceptions of human dance such that people who associate elements of a dance with desirable personality characteristics and—as a consequence—rate that dance as more attractive. In a classic study by Heider and Simmel (1944) participants viewed a brief movie showing the interaction of a rectangle, triangles and a circle and attributed intentional movement and goal-directed interactions to these shapes although no social cues were present. Although many interpretations have been offered over the years, it seems that basic features of these objects, together with their amplitude, speed, and velocity of movement are sufficient to cause personality attributions (see also Brownlow et al., 1997; Koppensteiner and Grammer, 2010, 2011). We suggest that future studies on relationships of objective (biomechanical) characteristics and perception of human body movement will arrive at similar conclusions.

In summary, humans attend to diverse stimuli to secure information about others, particularly when assessing attractiveness. There is robust evidence from the study of static representations of human faces and bodies that certain features provide information about an individual’s quality and this has consequences for human social perception, including people’s mating psychology and behavior. Recent research shows that quality information can also be obtained from body movement, such as dance and gait. Whether this information affords accurate assessment of personality characteristics is unclear. Preliminary evidence suggests that personality cannot be assessed accurately from dance movements, although people agree on trait attributions to dance movements. Thus, it may be that movement quality—as expressed by combination of amplitude, speed, and velocity—creates an impression about personality. Dance movements, however, seem to provide information about physical strength, especially in men, and women prefer the dances of men who are physically stronger. Comparative studies on motor performance in non-human animals indicate that elaborate and acrobatic courtship dances correlate with male reproductive success. For example, Barske et al. (2011) showed that female golden-collared manakins (*Manacus vitellinus*) preferred males that performed displays at greater speed. Reproductively successful males also had higher heart rates, which was interpreted as greater metabolic investment in courtship displays. It is premature to speculate on analogous phenomena in humans, although it is plausible that women may judge energetically demanding dance movements more positively, because such displays are produced by high quality men. If women consider dance movements in their assessments of male quality, this raises questions about the relative strengths of the effects of static and dynamic cues on attractiveness assessments. Studies on facial and bodily attractiveness show that impression formation is made rapidly, and although rapid trait attribution has been reported for movement too, movement cues may contribute to a more detailed assessment of a person than impression formation based on static cues. We therefore recommend that attractiveness research includes assessments of body movement.

### Conflict of Interest Statement

The authors declare that the research was conducted in the absence of any commercial or financial relationships that could be construed as a potential conflict of interest.

## Footnotes

1 We omit reference to data on dance and symmetry, as reported by Brown et al. (2005). Careful investigation revealed that some of the study data were fabricated and a re-analysis could not confirm the reported results (Trivers et al., 2009). The Brown et al. (2005) paper was retracted on November 27, 2013. For a more detailed discussion of the case of dance and symmetry see Fink et al. (2014).
